# Molecular Insights Into the Gating Kinetics of the Cardiac hERG Channel, Illuminated by Structure and Molecular Dynamics

**DOI:** 10.3389/fphar.2021.687007

**Published:** 2021-06-08

**Authors:** Zheng Zequn, Lian Jiangfang

**Affiliations:** ^1^ Department of Cardiovascular, Medical College, Ningbo University, Ningbo, China; ^2^ Department of Cardiovascular, Lihuili Hospital Affiliated to Ningbo University, Ningbo, China

**Keywords:** HERG potassium channel, cryo-electron microscopy structure, molecular dynamics simulations, voltage gating regulation, molecular mechanisms

## Abstract

The rapidly activating delayed rectifier K^+^ current generated by the cardiac hERG potassium channel encoded by *KCNH2* is the most important reserve current for cardiac repolarization. The unique inward rectification characteristics of the hERG channel depend on the gating regulation, which involves crucial structural domains and key single amino acid residues in the full-length hERG channel. Identifying critical molecules involved in the regulation of gating kinetics for the hERG channel requires high-resolution structures and molecular dynamics simulation models. Based on the latest progress in hERG structure and molecular dynamics simulation research, summarizing the molecules involved in the changes in the channel state helps to elucidate the unique gating characteristics of the channel and the reason for its high affinity to cardiotoxic drugs. In this review, we aim to summarize the significant advances in understanding the voltage gating regulation of the hERG channel based on its structure obtained from cryo-electron microscopy and computer simulations, which reveal the critical roles of several specific structural domains and amino acid residues.

## Introduction

Since the identification of the *KCNH2* (hERG) gene in 1994, a multitude of studies have gradually revealed the significant role of the fast component of the inward delayed rectifier potassium current (I_Kr_) generated by the hERG potassium channel in the formation of the complete action potential (Curran et al., 1995; Perry et al., 2015). Following the identification of another slow component, I_Ks_, encoded by *KCNQ1/KCNE1*, the hook-shaped tail current from the hERG channel established this channel as the most critical myocardial repolarization reserve (Roden and Yang, 2005; Schmitt et al., 2014; Skinner et al., 2019). The full-length protein of the *KCNH2* gene contains 1,159 amino acids (Ono et al., 2020), and mutations at different positions that affect the voltage gating kinetics of the channel are classified as class III mutations (Delisle et al., 2004; Smith et al., 2016; Mehta et al., 2018; Schwartz et al., 2019). Unlike the class II mutations, which mainly lead to intracellular protein maturation defects, class III mutations in different domains of the hERG channel change its gating kinetics mainly manifesting as slowed channel activation or accelerated deactivation. Furthermore, it is well known that the hERG channel is the most common target for arrhythmogenic drugs, which can directly change its gating kinetics or affect the biogenesis of the channel protein. Dysfunction of the hERG channel caused by congenital gene mutations or drug blockade prolong the duration of the cardiac action potential, which may trigger fatal arrhythmia, such as torsades de pointes (TdP) (Sala et al., 2016; Smith et al., 2016; Mehta et al., 2018; Perry et al., 2020).

Due to the important role of the hERG channel in the formation of cardiac action potentials, exploring the key factors that determine the gating kinetics of the channel and the protein domains involved in gating regulation is expected to suggest therapeutic strategies to address hERG channel dysfunction and its negative gating changes, thus preventing potential severe arrhythmia. However, hERG channel itself is a complicated tetramer structure composed of four subunits with other auxiliary subunits, and site-directed mutagenesis studies have shown that almost every domain located in the full-length channel protein is involved in its gating regulation. These factors affect the development of hERG channel gating regulation drugs. Because of the complicated interactions between the different channel domains, identifying the normal voltage gating mechanism of the hERG channel during myocardial repolarization requires an improved understanding of the role of the involved domains in a conformational change of the channel. Therefore, the high-resolution channel structure on the cell membrane, which shows the location of key regulatory domains, may contribute to the analysis of the gating characteristics of the hERG channel and help us to understand its importance in the action potential.

The Shaker channel, another voltage-gated K^+^ (VGK) channel, has provided a basic understanding of the structure and dynamics of the classic cardiac VGK channels (Barros et al., 2019). In addition, site-directed mutagenesis studies have identified important structural domains that regulate the conformational changes of the hERG channel and high-affinity sites for drug binding (Mitcheson et al., 2000; Chen et al., 2002; Perry et al., 2004; Vandenberg et al., 2017). The structure of hERG recently obtained from cryo-electron microscopy, named hERG_T_, showed subtle structural differences. It provides new insights into the gating mechanism and reveals the molecular basis of drug hypersensitivity (Wang and MacKinnon, 2017) (Table 1). Developments in X-ray crystallography and cryo-electron microscopy have revealed the structures of these proteins look in atomic detail but do not tell us how they function. Molecular dynamics (MD) simulation, a powerful forecasting tools, has yielded mechanistic details of fundamental processes that are not available through experiments alone (DeMarco et al., 2019). It has progressed to the point that we can now simulate realistic molecular assemblies to produce quantitative calculations of the thermodynamic and kinetic quantities that control function, such as ion conduction and ion channel gating (Flood et al., 2019). Since the various domains of the hERG channel may be involved in the regulation of its conformational and functional changes, whether induced by congenital gene mutations or drugs, only by combining structural and functional studies of site-directed mutations with MD simulations, etc. can we better understand the underlying complex kinetics (Schewe et al., 2019; Dickson et al., 2020).

**TABLE 1:** Summary of the main structure acquisition and molecular dynamics simulation results mentioned in the paper.

Aim	Methods	Softs or programs	Parameters	Results	Limitation	References
To characterize the high-resolution hERG structure	Cryo-electron microscopy (Cryo-EM)	***Modeling*** Blocres, Bsoft RELION, Frealign ***Generation of atomic model*** HOLE, COOT	***Channel*** hERG channel (hERG_T_ and hERG_Ts_) ***Force field*** AMBER	The cryo-EM structure of hERG channel-hERG_T_ of 3.8 Å and its non-inactivating mutant hERG_Ts_ S631A	Low recognition of intracellular structural domains and unable to clarify the mechanism of non-domain exchange	Wang and MacKinnon (2017)
To characterize the structural properties of the cNBD^a^ in KCNH2	SEC-MALS^b^ SAXS^c^ Electrophysiologica-l recording	***Modeling*** PHASER, COOT ***SEC-MALSS*** Astra ***SAXS*** PRIMUS, CRYSOL	***Channel*** hERG channel ***Force field*** -	cNBD of 1.5 Å and a novel salt-bridge E807-R863	No simulation of the function of the salt bridge in different VGK channel^d^	Ben-Bassat et al. (2020)
To explore the VSD/PD^e^ coupling mechanism in VGK channels	GIA^f^ Network analysis of MD^g^	***Image building*** VMD^h^ ***MD*** Carma, Python	***Channel*** Kv1.2/2.1 chimera ***Force field*** -	S4–S5 linker regulates the position of the S6 gates in the Kv1.2/2.1	Unable to accurately estimate the charge of each channel	Fernández-Mariño et al. (2018)
To identify the domains involving the passage of ions through VGK channels	MD Electrophysiologica-l recording	***MD*** CHARMMc36 ***Electro-physiological*** Clampfit 10	***Channel*** KcsA channel ***Force field*** CHARMM	Ligand-induced conformational changes in the KcsA channel removes steric restraints at the SF^i^	No comparison of differences in different penetration mechanisms under different force fields	Heer et al. (2017)
To identify activators that open different potassium channels	X-ray crystallography Cysteine-scanning mutagenesis Atomistic MD	***Crystallography*** **XDS** XSCALE/AIMLESS ***Atomistic MD*** Glide, GROMACS 5.0 and 5.1	***Channel*** K2P channels, VGK BK_Ca_ channels^j^ ***Force field*** generalized amber force field (GAFF)	A class of negatively charged activators (NCAs) act as master keys to open K^+^ channels gated at their SF	The differences between various potassium channels may challenge this channel opening mechanism	Schewe et al. (2019)
To clarify the mechanism of the passage of ions through VGK channels	X-ray crystallography Extensive molecular MD	***Crystallography*** HKL 2000, PHASER COOT, PHENIX ***MD*** GROMACS 5.1	***Channel*** MthK channel ***Force field*** CHARMM36 AMBER	Channel conductance is controlled at the SF	The simulations go beyond the openings of channel observed in the experiment	Kopec et al. (2019)
To quantitatively elucidate the thermodynamic basis for the AG/SF coupling	Adiabatic energy maps FEP/MD^k^	***Atomic model*** ***Construction and MD*** CHARMM	***Channel*** KcsA channel ***Force field*** CHARMM	Phe103 as the critical residue controlling the allosteric AG/SF coupling	FEP/MD only analyzed a single site-directed mutation in detail	Pan et al. (2011)
To understand molecular origins of gating shifts	Solid-state- NMR^l^ MD	***NMR*** PISSARROSPARTA ***MD*** NAMD 2.11, Anton	***Channel*** KcsA channel ***Force field*** CHARMM	E71X mutations change the equilibrium between intrinsically sampled filter states	No further experiments to comprehend C-type inactivation	Jekhmane et al. (2019)
To characterize a structural basis for the inactivation mechanism	Homology modeling MD	***Modeling*** Modeller 7v7, pymol ***MD*** Gromacs 3.2	***Channel*** hERG channel ***Force field*** GROMOS96 43a1	Phe627 play a significant role in the hERG C-type inactivation	Shorter MD simulation time cannot replicate the inactivation process of the hERG channel	Stansfeld et al. (2008)
To understand the molecular mechanism of C-type inactivation	MD Electrophysiologica-l recording	***MD*** VMD, NAMD 2.12 NAMD 2.13, Anton ***Electrophysiolog-y*** GPatch	***Channel*** hERG channel ***Ensemble*** NPT ***Force field*** CHARMM	The constricted conformation in hERG involves the residues Y616, N629 and F627	MD still cannot completely solve the unknown molecular mechanism of complex channel regulation	Li et al. (2021)
To simulate specific interactions between VSD and PD	Homology modeling MD	***Modeling*** ClustalW2, TMPred ***MD*** ROMACS 4.5.3	***Channel*** hERG channel ***Force field*** OPLS all-atom	L532 as a structural basis for hERG channel deactivation	The simulation times in fully atomistic models cannot to explore large-scale movement involving	Colenso et al. (2013)
To understand the mechanism of voltage gating in VGK channel	All-atom MD	***MD*** Anton, HiMach	***Channel*** Kv1.2/2.1 chimera ***Ensemble*** NPT ***Force field*** CHARMM27	To show how a VGK channel switches between activated and deactivated states	The relatively slow calculation speed leads to the system disequilibrium	Jensen et al. (2012)

a cNBD: C-terminal cyclic nucleotide binding domain

b SEC-MALS: size-exclusion chromatography combined with multi-angle light scattering

c SAXS: small-angle x-ray scattering

d VGK channels: voltage-gated K+ channels

e VSD: voltage sensing domain, PD: pore domain

f GIA: Generalized Interaction Energy Analysis

g MD: molecular dynamics

h VMD: Visual Molecular Dynamics

i SF: selectivity filter

j BKCa channels: Ca2+-activated BK-type channels

k FEP/MD: free energy perturbation/molecular dynamics

l NMR: Nuclear Magnetic Resonance Spectroscopy, “-” represents “not given”

For new insights into the structure and kinetics, it is hoped that identifying the key residues that determine the different conformations of the channel and achieving a new understanding of structural differences in hERG_T_ will enable the correction of hERG channel dysfunction. More importantly, by further identifying which components affect channel gating and how, based on the ability to see the atomic structure inside the ion channel, reversing the dysfunction may require only targeting a single residue located in the channel. Therefore, understanding the molecular level of ion channel physiology has great biomedical significance.

## The Most Important Repolarization Reserve

The cardiac hERG channel, also known as the Kv11.1 channel or ERG1 potassium channel, is one of the ether-a-go-go (EAG) potassium channel family members (Barros et al., 2020; He et al., 2020). The proposed sequence of the biosynthesis of hERG channel protein is translation, insertion into the endoplasmic reticulum membrane, the addition of asparagine-linked (N-linked) glycans, the tetramerization of α-subunits, and the correct or native folding of the voltage sensor, pore, NH2 terminus, and COOH terminus (Ono et al., 2020). Mature channel proteins in the membrane become the most critical myocardial repolarization reserve depending on the characteristics of the inward delayed rectifier, which facilitates the completion of depolarization and ensures a normal action potential duration (Roden and Yang, 2005; Skinner et al., 2019).

In addition to congenital mutations that lead to functional loss of the channel, the off-target effects of many drugs target hERG channel to cause current reduction, and the resulting cardiotoxicity is sometimes fatal due to the specific role of the hERG channel in the heart (van Noord et al., 2010). Dysfunctional mutation of the hERG gene causes haplotype deficiency or dominant-negative effects in its protein expression, which can partially or completely reduce the I_Kr_ current and thus prolong the repolarization time course (Egashira et al., 2012; Zhang et al., 2014; Cubeddu, 2016; Ono et al., 2020). Similarly, cardiotoxic drugs bind directly to highly sensitive aromatic amino acid residues (Y652, F656, etc.) in the channel that have a direct blocking effect or prevent the mature expression of channel proteins on the membrane (Vandenberg et al., 2017; Helliwell et al., 2018; Zequn et al., 2021). For example, the antimalarial drugs quinidine and diastereoisomers of quinine show stereoselectivity for the hERG channel, with quinine 14-fold less potent than quinidine, and the hERG-F656C mutation reverses this stereoselectivity, suggesting that residue F656 contributes to the stereoselective pocket for quinidine and quinine (Yan et al., 2016).

**FIGURE 1:**
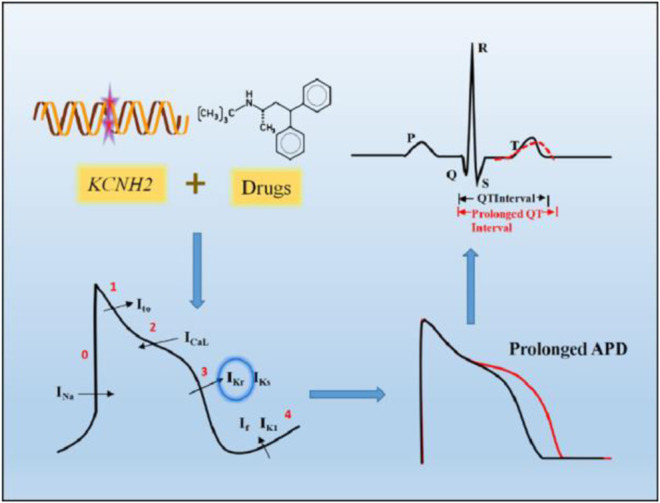
Prolonged QT interval on ECG caused by hERG channel dysfunction. *KCNH2* gene mutations and drugs targeting hERG channel can partially or completely reduce I_Kr_, which mainly function in the third phase of cardiac action potentials, resulting in prolonged action potential duration (APD), shown on the ECG as a prolonged QT interval.

The effects of both gene mutations and toxic drugs targeting the hERG channel can be expressed as the QT interval of the electrocardiogram (ECG), and prolongation of the duration beyond the normal range (440 ms for men and 460 ms for women) is clinically called long QT syndrome (LQTS) (van Noord et al., 2010; Schwartz and Ackerman, 2013) (Figure 1). Because of the consequent reduced repolarization reserve, excessive prolongation of the QT interval may trigger early after depolarization (EAD) and even induce malignant arrhythmias, such as TdP and ventricular fibrillation (Schwartz & Woosley, 2016).

## Structural Differences From hERG_T_

**FIGURE 2:**
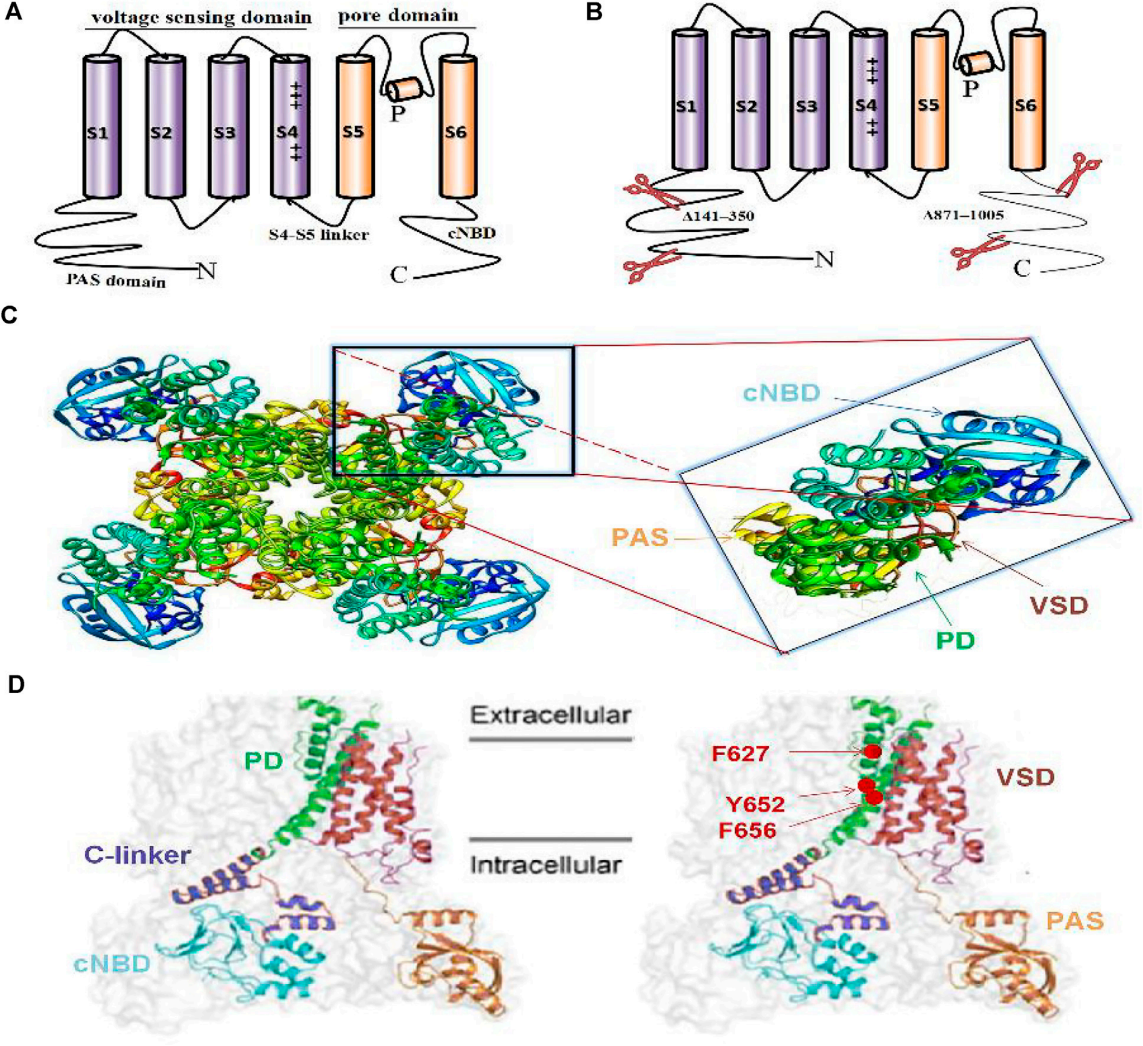
Single alpha subunit of the previous hERG structure **(A)**, new hERG_T_ structure **(B)**, 3D side view of full hERG_T_ **(C)**, obtained from PDB: 5VA2; hERG_T_) and single subunit of the hERG_T_ on the cell membrane **(D)**. The classic VGK channel structure contains six transmembrane segments S1-S6, S1-S4 contains charges to form the voltage-sensing domain (VSD), and S5-S6 forms the pore domain (PD) **(A)**. Cryo-electron microscope (cryo-EM) structure hERG_T_ with deletion of the N-terminal 141–350 and the C-terminal 871–1,005 of the non-structural sequence to prevent aggregation during purification **(B)**. Full-length hERG_T_ channel contains four subunits arranged in a circular pattern around a central PD (C-left) and magnifying single subunit, colored to distinguish different domains (C-right). The color-marked single subunit corresponding to the above 3D structure on the cell membrane, including the PAS domain at the N-terminal and the C-terminal cyclic nucleotide binding domain (cNBD), transmembrane PD and VSD, as well as the main blocking sites Y652, F656 for specific cardiotoxicity drugs and F627 involving inactivation **(D)**.

The hERG channel shares structural homology with other VGK channels (Whicher and MacKinnon, 2016; Vandenberg et al., 2012; Dehghani-Samani et al., 2019). Within contrast to Na^+^ channels, the four hERG subunits are cyclically rather than linearly arranged on the membrane to form a tetramer (Vandenberg et al., 2012; Brewer et al., 2020). The hERG channel tetramer is assembled in the endoplasmic reticulum with the help of the human J-protein chaperones DNAJB12 and DNAJB14 (Li et al., 2017). On the membrane, each subunit contains six transmembrane fragments (S1-S6), an N-terminal Per-Arnt-Sim (PAS) domain, and a C-terminal cyclic nucleotide binding domain (cNBD) (Barros et al., 2020; Vandenberg et al., 2012; Shi et al., 2020). S1-S4, as the voltage-sensing domain (VSD), possesses multiple positively charged residues to sense changes in the transmembrane voltage, and which S4 is the most important (Dehghani-Samani et al., 2019; Barros et al., 2012). S1-S3 contain some negatively charged amino acids, which are considered to form the offset charge of the entire VSD. S5-S6 are important structures that constitute the channel pore domain (PD). They form a tetrameric structure surrounding the central conduction pathway by coupling with the middle pore ring of the four subunits and constitute the ion-permeable PD (Barros et al., 2020; Wang et al., 2011). The connection between S4 and S5, the S4-S5 linker, transmits the voltage change information from the VSD to the PD, bridging or translating from the voltage change to physical action (Dehghani-Samani et al., 2019) (Figure 2A). It has been reported that the eag domain, which contains the PAS domain and the PAS-Cap domain at the N-terminus, is essential in stabilizing the assembly and trafficking of the hERG subunits (Ke et al., 2014). In addition, mutations at different positions in the cNBD impair the interaction with the eag domain and accelerate the deactivation of the channel (Kume et al., 2018).

Recently, the cryo-EM structure of voltage-gated potassium channels has attracted great attention for its great contribution to revealing the gating mechanism and reasons for drug hypersensitivity. The hERG homologous family EAG1 (Kv10.1) structure, which removed 114 residues (773–886) from the unstructured C-terminus to achieve a resolution of 3.78 Å, is called rEag1Δ and retains all the characteristics of the full-length channel, including similar gating kinetics (Whicher and MacKinnon, 2016). Subsequently, the hERG cryo-EM structure was obtained: hERG_T_ was constructed by the deletion of two fragments (N-terminal 141–350 and C-terminal 871–1,005), and the remaining 814 amino acid residues also have similar gating characteristics to the wild-type hERG channel (hERG_WT_) (Wang and MacKinnon, 2017) (Figures 2B–C–D). The hERG_T_ structure has a resolution as high as 3.8 Å and shows subtle differences from the hERG_WT_. First, its S4-S5 linker is the same as the rEag1Δ structure, which is only a short loop containing five residues, not as long as previously thought (Wang and MacKinnon, 2017). Second, unlike the Shaker Kv channel with six or more charged residues, the S4 helix has only five positively charged amino acids, and the first three, K1, R2, and R3, are located outside the cell, while R4 and R5 are inside, indicating that the VSD is in an active state of depolarization (Brewer et al., 2020). In addition, unlike the EAG1 channel, whose pore is closed owing to the presence of Ca^2+^ and calmodulin, the pore in hERG_T_ is open, and the volume of the central cavity of the channel is very small (Whicher and MacKinnon, 2016; Wang and MacKinnon, 2017; Butler et al., 2019).

Notably, there are four deep and elongated hydrophobic pockets stretching into the central cavity to form the selectivity filter (SF), and the drug-sensitive amino acids are located on the surface of these pockets. The well-known hERG channel blockers astemizole and dofetilide embed in the pocket after entering the narrow central cavity. The exposed hydrophobic pocket clearly provides a unique location for drug binding. Furthermore, an extreme negative electrostatic potential is caused by the smaller volume of the central cavity, making it attractive to channel blockers, which contain mainly positive charges, which may be useful to explain the abnormal drug sensitivity of the hERG channel (Wang and MacKinnon, 2017; Butler et al., 2019).

The cryo-EM structure provides groundbreaking insights into the complete channel structure, but its atomic-resolution details of the intrinsic structural domain of the channel in the cytoplasm are limited. The high-resolution crystal structure of the first hERG channel with a resolution of 1.5 Å identified a functionally vital salt bridge (E807-R863), which did not appear in the cryo-EM structure. Electrophysiological analysis indicated that the salt bridge may not only support the spatial organization of the internal ligands but also maintain the complex interface within the cell (Ben-Bassat et al., 2020) (Table 1).

## Voltage Gating of the hERG Channel

### Gating Kinetics in Cardiac Action Potential

**FIGURE 3:**
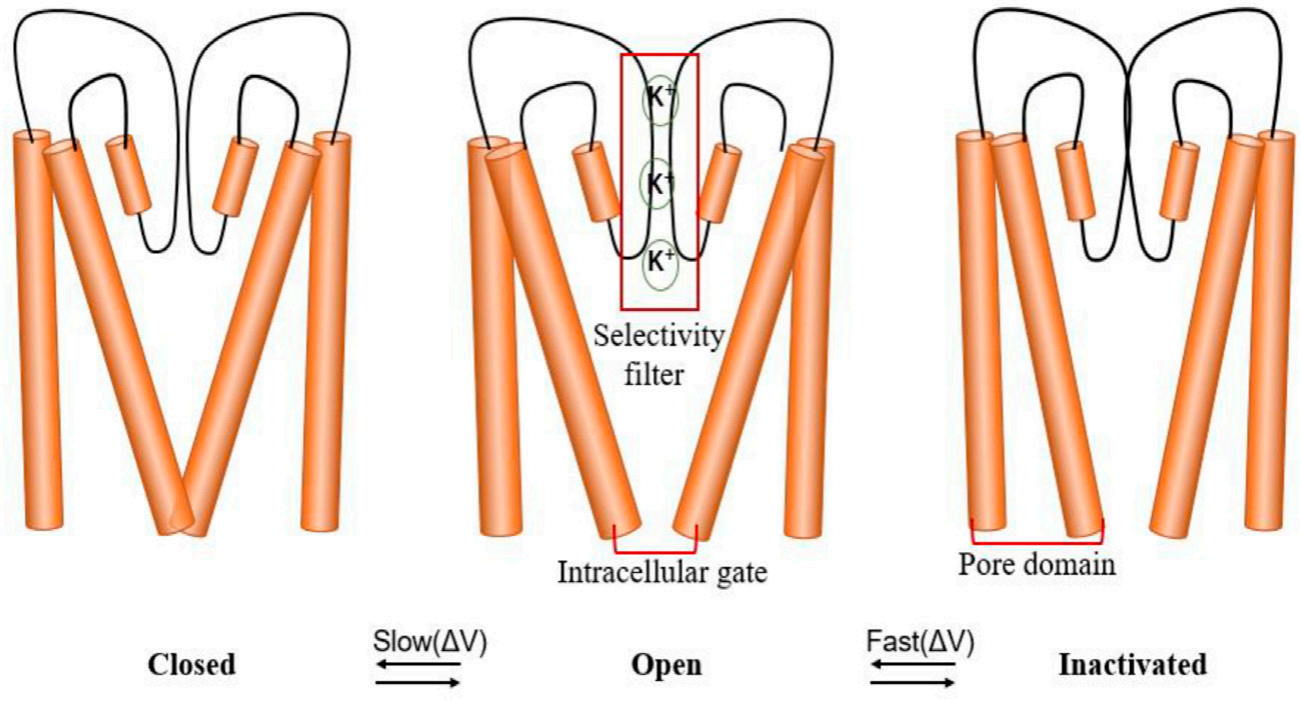
hERG channel gating. The channel has three states: closed, open and inactivated. Schematic diagram of the hERG channel gating scheme, indicating that the state transition between closed and open states is slower than the state transition between open and inactive states. The selectivity filter (SF), located in the central cavity of the pore domain, and the intracellular activation gate (AG) coupling (AG/SF coupling) are thought to participate in different state changes.

Similar to other VGK channels, the hERG channel has three different conformations: closed, open, and inactivated (Vandenberg et al., 2012; Perry et al., 2015; Vandenberg et al., 2017; Zhang et al., 2018; Shi et al., 2020). However, hERG also has unique gating kinetics, that is, rapid inactivation and recovery from inactivation, while activation and deactivation are relatively slow (Vandenberg et al., 2017; Shi et al., 2020). In other words, the inactivation of the hERG channel is more active than the activation, contributing to its inward rectification characteristics (Smith et al., 1996; Spector et al., 1996) (Figure 3).

In the early stage of the action potential, the hERG channel opens slowly but inactivates quickly. As the depolarization voltage continues to decrease, the channel gradually recovers from inactivation (Witchel et al., 2001). When the membrane potential is −40 mV, the transmembrane current reaches its peak value. The I_Kr_ current gradually increases during the repolarization process, followed by slow deactivation of the channel (Vandenberg et al., 2012). The channel thus undergoes activation, inactivation, reactivation, and deactivation during the entire course of the action potential, and the channel remains open for a long period (200–300 ms) below the resting potential, resulting in a typical tail current (Barros et al., 2012; Vandenberg et al., 2017).

### Key Molecules Involved in the Regulation of Gating Kinetics

**FIGURE 4:**
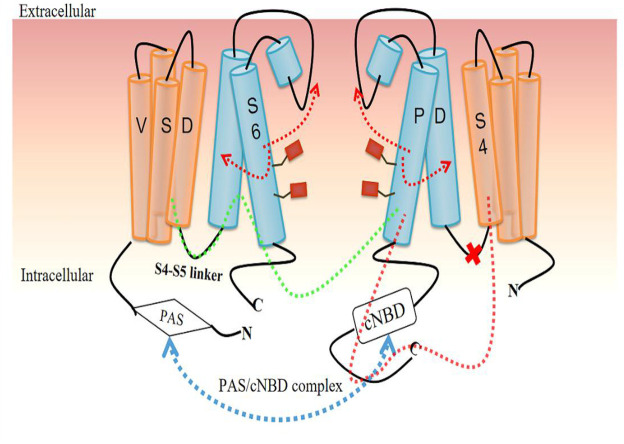
Voltage gating regulation model for hERG channel based on the PAS/cNBD complex. The PAS/cNBD complex plays a central role (*blue dotted line*) in this model. Previously, the S4-S5 linker was thought to act as a bridge to transform the conformational changes in the voltage-sensing domain (VSD) into physical movement of the pore domain (PD) and then interact with adjacent subunits to regulate the pore gate (*green dotted line*). A new insight is that the S4-S5 linker cannot act as a bridge (*red cross*), but S4 of VSD directly interacts with S6 of the same subunit, resulting in a change in the state of S6 followed by opening or closing of the channel gate (*red dotted line and arrow*).

The regulation of different conformational changes of hERG channels is so complicated that it is difficult to elucidate the exact mechanism of gating kinetics because almost all domains are involved in this regulation (Codding et al., 2020). Some domains at the C-terminus and N-terminus, particularly the PAS domain and cNBD, have been proven to play key roles in the regulatory mechanism. They may affect the interaction between the PAS domain in one subunit and the cNBD in an adjacent subunit, called the PAS/cNBD complex (Codding et al., 2020; Morais-Cabral and Robertson, 2015; Adaixo et al., 2013) (Figure 4). This physical interaction between the different domains of different subunits can produce electrical activity through further coupling, which is a classic explanation for the changes in different conformational states of VGK channels. However, this electromechanical coupling model has been questioned in recent studies (Wang and MacKinnon, 2017; Barros et al., 2019; Butler et al., 2019; Jekhmane et al., 2019; Kopec et al., 2019; Malak et al., 2019).

Homology modeling approaches suppose that protein structure is more conserved than protein sequence. This is usually the case for ion channels, where relatively low 30–40% sequence similarities are accompanied by strong structural resemblance, deviating only by a few angstroms (Forrest et al., 2006). New structural differences from hERG_T_ and homology models such as KcsA, a prokaryotic channel lacking a VSD that provides an excellent prototypical model system to understand the kinetics of VGK channels, have enabled MD simulations to gradually identify the roles of important gated regulatory molecules.

#### S4-S5 Linker and Selectivity Filter in Activation

As shown above, the activation process of the hERG channel is very slow, with a voltage threshold of −40 mV to −30 mV, reaching a plateau at +20 mV to +40 mV (He et al., 2020). The physiological significance of slow activation is to minimize the use of channels to reduce antagonism with the inward ion flow of Na^+^ and Ca^2+^(Shi et al., 2020). It is generally believed that the structural rearrangement in VSD is transferred to PD through the physical interaction between the S4-S5 linker and the cytoplasmic end of the S6 helix, opening and closing the activation gate. This conversion of energy from voltage to mechanical motion is called the VSD/PD coupling mechanism (Barros et al., 2020; Shi et al., 2020; Hull et al., 2014; Tristani-Firouzi et al., 2002; Kalstrup and Blunck, 2018) (Figure 4). The foundation of this model is that the positively charged residues of S4 move due to the force applied by the transmembrane electric field and become the driving force for the conformational changes in the pore region (Jensen et al., 2012).

Therefore, the existence of the S4-S5 linker as a bridge seems to be necessary for the interaction in different domains to produce conformation change. Additionally, the slow movement of S4 may explain the slow activation. In a recent study combining experiment and MD analysis, a rack‐and‐pinion type of coupling between VSD and PD involving interactions between helices S4 and S5 was suggested to be a dominant activation process and an alternative mechanism for VGK channels in the Shaker family (Fernández-Mariño et al., 2018, Table 1). However, cutting off the S4-S5 linker and thus breaking the connection between VSD and PD did not change the activation kinetics of the channel, indicating that a bridge may not be needed to induce pore opening (Lörinczi et al., 2015; de la Peña et al., 2018) (Figure 4). Another piece of supporting evidence arises from the incorporation of fluorescent unnatural amino acid tags at different positions of the S4-S5 linker, which showed that the movement of the linker is not related to the movement of S6, but the channel still opens normally (Kalstrup and Blunck, 2018).

The hERG_T_ structure also calls the VSD/PD model into question. First, the S4-S5 linker is a short loop containing five residues that cannot act as a mechanical lever to perform domain exchange. Second, the S6 segment lacks the proline-valine-proline (PVP) motif that is considered, in other VGK channels, to narrow the pore region to allow S6 to interact with the S4–S5 linker. Thus, the interaction between VSD and the C-terminus of the S6 segment occurs in the same subunit and does not involve another adjacent subunit (Wang and MacKinnon, 2017). Compared with other VGK channels, the structure of this non-domain exchange shows differences in the movement of the VSD as the mechanism of channel opening (Malak et al., 2017; Malak et al., 2019). Based on this new structural difference, researchers proposed another mode of VSD/PD, showing that S4 directly interacts with the C-terminal joint to cause the bending and relaxation of S6 without the need for the S4-S5 linker (Wang and MacKinnon, 2017). The bending and relaxation of S6 are caused by the different states of S4. When the VSD is in a depolarized state, the S4-S5 linker interacts with the intracellular part of S6 to guide the C-terminus of S4 to the C-terminal joint to relax S6 and open the channel. In contrast, the hyperpolarized state of the VSD causes S6 to bend and thus close the pore gate (Wang and MacKinnon, 2017; Butler et al., 2019) (Figure 4).

In summary, the VSD/PD coupling mechanism cannot simply explain the slow activation of the channel. Although the new model provides a possible direction, the exact relationship between the conformational state of VSD and the activation gate remains to be confirmed. Critically, cryo-EM usually has a low resolution for the periphery of the protein structure, which hinders the clear identification of side-chain conformations and the accurate modeling of channel functional characteristics (Herzik et al., 2019). Nonetheless, docking directly to this cryo-EM structure has been reported to yield binding modes that are unable to explain known mutagenesis data. Therefore, further MD simulation may be an effective tool to support the above hypothesis and produce consistent data.

In potassium channels, predominately in the context of C-type inactivation, the allosteric coupling between the activation gate (AG) and the SF has been studied for a long time. This coupling was proposed to play a role in the closed-to-open transition, where upon initial opening, the AG affects the SF by changing its conformation from prime-to-conduct to conductive (Heer et al., 2017) (Figure 3). MD simulations and electrophysiology measurements showed that ligand-induced conformational changes in the KcsA channel remove steric restraints at the SF, resulting in structural fluctuations, reduced K^+^ affinity, and increased ion permeation. Such activation of the SF may be a universal gating mechanism within VGK channels (Heer et al., 2017, Table 1).

More recently, a class of negatively charged activators (NCAs) have been reported to act as master keys to open potassium channels (K2P, BK, and hERG) gated at the SF via a conserved but not specific mechanism. Through functional analysis, X-ray crystallography, and MD simulations, the NCAs were shown to bind to similar sites below the SF, increase pore and SF K^+^ occupancy, and open the filter gate. In addition, MD simulations of the calcium-gated prokaryotic potassium channel MthK provide further evidence to support AG/SF coupling. Therefore, several structurally distinct potassium channels were postulated to be activated/gated at the SF (Schewe et al., 2019, Table 1). The motions of the pore-lining TM helices constituting the AG underlie the narrowing and widening motions of the SF, predominately at residue T59, the equivalent threonine in KcsA, which in turn controls the ionic flow through the channel (Kopec et al., 2019, Table 1). In summary, the channel’s conductance is regulated at the SF through allosteric coupling with the AG (Labro et al., 2018). Thus, targeting a specific residue in the SF to design activators of the hERG channel may reverse the steric hindrance effect of drugs that bind directly to the channel.

#### Selectivity Filter and F627 in Inactivation

After the hERG channel is slowly activated, an abnormally rapid inactivation process occurs, and the channel can be inactivated within 1–2 ms at +60 mV (Shi et al., 2020). Rapid inactivation and recovery from inactivation maintain the steady state of the action potential (Perry et al., 2015). The inactivation of the hERG channel is essentially of P/C type because it is not affected by N-terminal deletion (Schönherr and Heinemann, 1996). This type of inactivation is sensitive to the ion occupancy in the SF, indicating that inactivation may involve the structural rearrangement of the SF (Schönherr and Heinemann, 1996). A selective and rapid flux of K^+^ across the cell membrane through a central pore is regulated by the interplay between an AG and a C-type inactivation gate known as SF, called activation coupled to C-type inactivation (Tilegenova et al., 2017; Jekhmane et al., 2019; Kopec et al., 2019).

MD simulations of the pore helix and SF regions of the hERG channel based on KcsA have provided insights into the molecular basis of inactivation (LeMasurier et al., 2001). Using free energy perturbation MD simulations, Phe103, a residue located along the inner helix, was identified as the critical residue controlling the allosteric coupling between SF and AG (Pan et al., 2011, Table 1). Furthermore, T75A in KcsA proves that, upon activation, the SF transitions from a nonconductive and deep C-type inactivated conformation to a conductive conformation (Labro et al., 2018).

In VGK channels, the term mode shift or hysteresis determines a normal heartbeat and regulates cell excitability (Männikkö et al., 2005; Corbin-Leftwich et al., 2016). One example of the physiological importance of hysteresis is in the regulation of the activation, deactivation, and inactivation gating of voltage-gated Na^+^ channels and VGK channels (Villalba-Galea, 2017). Initially, the time course of development of the mode shift was correlated with that of P/C-type inactivation, suggesting that P/C-type inactivation is required for mode shift to occur (Olcese et al., 1997). Hysteresis in tetrameric cation-selective channels can arise from AG/SF allosteric coupling, and mode shift was prevented in the total absence of C-type inactivation, indicating that SF plays an essential role in activation-inactivation coupling (Tilegenova et al., 2017) (Figure 3). By integrating solid-state NMR and MD simulations, the E71 point mutation E71X was shown to rearrange the network behind the SF and perturb the K^+^ binding sites V76 and Y78, thus changing the equilibrium between the intrinsically sampled filter states (Jekhmane et al., 2019, Table 1). Interestingly, the Y78 conformation can change in reference to the filter mode, which is consistent with a recent cryo-EM structure of the hERG channel (Cheng et al., 2011; Wang and MacKinnon, 2017).

Unlike other VGK channels, the SF part of the hERG channel located on PD is not conserved and has some slight sequence differences. Five residues in KcsA, Trp67, Trp68, Glu71, Tyr78, and Asp80, have been proposed to be involved in hydrogen bond networks that stabilize the structure of the SF in the inactivated states. The equivalent residues in the hERG channel are Tyr616, Phe617, Ser620, Phe627, and Asn629, and none of them is conserved (Vandenberg et al., 2012). Notably, C-type inactivation is governed by a complex hydrogen bond network behind the SF (Cordero-Morales et al., 2006). The variable residue-induced lack of hydrogen bond networks in the channel contributes to the greater inclination of the SF to “collapse”, leading to inactivation (Xu and McDermott, 2019). Specific mutations of SF residues may change the inactivation characteristics of hERG channels (Schönherr and Heinemann, 1996; Wang et al., 2011).

In MD simulations, several residues in the SF were displaced in the pore axis, especially F627 in the GFG motif (Stansfeld et al., 2008, Table 1). The positional specificity of F627 was confirmed under the cryo-EM structure. Compared to the ERG1 channel, the direction of F627 in hERG_T_ is offset. A non-inactivated S631A mutant structure hERG_Ts_ S631A to simulate the non-transient inactivation of other Kv channels showed that the position of F627 in the SF is similar to that in the other channels (Whicher and MacKinnon, 2016; Wang and MacKinnon, 2017). The unique position of F627 represents the subtle differences in the SF conformation, suggesting that hERG may be rapidly inactivated by adjusting a single residue. At the same time, the great subtlety of the conformational change of SF is also emphasized. When potassium ions are observed to flow out of the SF, the changes are different from the larger conformational changes of KcsA K^+^ channels (Brewer et al., 2020). The latest results from a long-time scale MD support the key role of SF and F627, which demonstrates that the asymmetrical constricted-like conformation of the SF and the side chain rotation of F627 and the hydrogen bond between Y616 and N629 are key determinants for the a C-type inactivation (Li et al., 2021, Table 1). More structural and simulation evidence is needed to further support the regulation of hERG channel inactivation through a single residue, which is of utmost significance for research on inhibitors that accelerate channel inactivation.

#### Voltage-Sensing Domain in Deactivation

Following rapid recovery from inactivation, the hERG channel undergoes slow deactivation. Deactivation represents the closing of the hERG channel and is an important landmark feature. The regulation of deactivation is so complicated that it cannot be explained by the slow movement of the VSD, because it involves multiple regions of the channel. The interaction between the N-terminal Cap domain (N-Cap), PAS, and cNBD is critical for regulating slow deactivation (Muskett et al., 2011; Ng et al., 2011; Gianulis et al., 2013). The mutation of N-Cap accelerates the deactivation of the channel (Muskett et al., 2011). The application of a PAS domain fragment (1–135) restores slow deactivation to N-terminally deleted (Δ2–354) fast-deactivating hERG channels (Gustina and Trudeau, 2012; Gustina and Trudeau, 2013). And mutations at different positions in the cNBD impair the interaction with the eag domain and accelerate the deactivation of the channel (Kume et al., 2018). These results indicate that the PAS/cNBD complex occupies a central position in the regulation of deactivation, and the eag domain containing N-Cap and PAS is particularly prominent.

Consistently, the hERG_T_ structure further proves that the N-terminus is indeed integrated on the VSD/PD interface, indicating that the N-terminal PAS domain may move to the plasma membrane during the slow deactivation process, altering the VSD/PD interaction (Barros et al., 2019). It is worth mentioning that in a study of HEK293 cells expressing wild-type (WT) or hERG_T_ channels, the deactivation rate was significantly slower for hERG_T_ cells whose two cytoplasmic regions were deleted, which may reflect the influence of changes to electrostatic interactions on the VSD (Zhang et al., 2020).

The pairwise comparison of wild-type and mutant channel models is considered to be a useful method to explain the uncertain functional data in the model structure. In this approach, a 0.5 μs MD simulation indicated that the hERG-L532P mutation reduced the extent of interaction across the S4-S5 interface, suggesting a structural basis for the greatly enhanced deactivation rate (Colenso et al., 2013, Table 1). All-atom MD simulations showed how a VGK switches between activated and deactivated states. Upon deactivation, pore hydrophobic collapse rapidly halts ion flow. Subsequent VSD relaxation, including a 15 Å inward S4 helix motion, completes the transition (Jensen et al., 2012, Table 1). Future MD simulations will likely be used to assess the validity of these assumptions.

**FIGURE 5:**
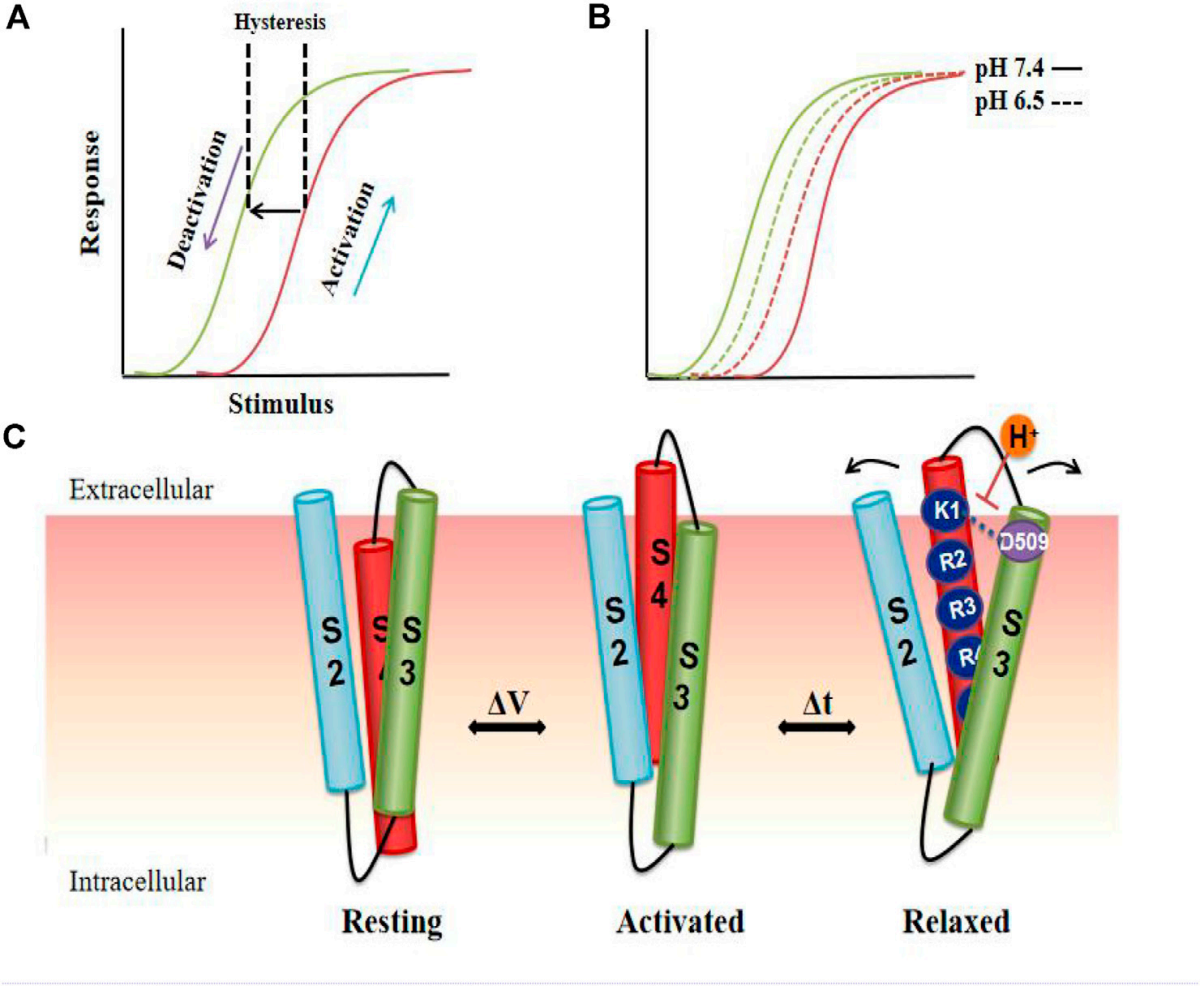
Model of VSD relaxation. In the hERG channel, depolarization activates the channel, and then repolarization causes the channel to deactivate and the VSD to enter a relaxed state, resulting in hysteresis. **(A)** Compared to pH 7.4 (*solid line*), the acidic pH (*dotted line*) shortens the hysteresis behavior of the channel, thereby accelerating the deactivation process. **(B)**. Cartoon representation of proposed reconfigurations of S2, S3, and S4 transmembrane domains in the resting, activated, and relaxed states. S4 in VSD undergoes a conformational change from a stable resting state to a relaxed state due to the voltage change. Putative reconfigurations associated with entering a relaxed state may involve the interaction of D509 with the positive external charge of S4. The neutralization of D509 or external protonation is predicted to destabilize the interactions, thereby destroying the relaxed state. Arrows indicate the hypothetical structural rearrangements of the helix during the relaxation process resulting from the interaction of the acidic residues in S2-S3 **(C)**.

As mentioned above, hysteresis can be observed in voltage-gated ion channels during each activation and deactivation cycle. The terms hysteresis and mode shift are often used to describe the separation between VGK channel activation and deactivation. Similar hysteresis behavior has been reported in a variety of potassium channels, such as KcSA (Tilegenova et al., 2017), Shaker (Labro et al., 2012; Priest et al., 2013), Kv3.1 (Labro et al., 2015), Kv7.2/7.3 (Corbin-Leftwich et al., 2016), Kv11.1 (hERG) (Goodchild et al., 2015; Shi et al., 2019) and Kv12.1 (Dierich et al., 2018). Relaxation of the VSD was identified as a reason for the slow deactivation of the hERG channel (Shi et al., 2020; Jensen et al., 2012; Shi et al., 2019). It is an inherent property of the VSD to be stabilized in a relaxed conformation after the hERG channel is activated. Relaxation causes energy separation of the channel activation and deactivation pathways, resulting in hysteresis and thus regulating the deactivation gating (Shi et al., 2019) (Figure 5). A dynamic model describing hERG channel VSD relaxation also shows that the instability of the VSD relaxation state, caused by extracellular protons, may drive voltage sensor return, leading to acceleration of the deactivation process (Shi et al., 2019; Wilson et al., 2019). In this model, acidic pH reduces the mode-shift behavior in hERG channels by destabilizing the relaxed state of the VSD, which is mediated by an extracellular acidic site, D509 (Figure 5). As an important contributor to slow deactivation, VSD relaxation could perhaps act as a master switch influenced by mutations within the N-terminus that stabilize the activated voltage sensor in a relaxed state (Goodchild et al., 2015; Thouta et al., 2017). Importantly, mode shift in the hERG channel occurs on a physiological time scale, suggesting that the voltage-dependent dynamic switching of activation and deactivation gating may contribute to the amplitude and timing of the repolarizing I_Kr_ current during the cardiac action potential.

Regulating the deactivation gating kinetics of hERG channels seems to be particularly important. Drugs that act as channel blockers or activators to alter this process by acting on the corresponding domain would produce an apparent pathogenic or therapeutic effect (Sala et al., 2016; Perry et al., 2020). Furthermore, developing more effective channel activators requires more detailed information about the role of the eag domain, VSD, and PD.

## Summary and Perspectives

There are a variety of potassium channels involved in regulating the action potential of cardiomyocytes, mainly including voltage-gated channels and ligand-gated channels. As a member of the VGK channels, the hERG channel has attracted much attention for its unique gating kinetics and its high affinity for cardiotoxic drugs. Early site-directed mutagenesis studies helped us identify which key amino acid in the hERG channel determines the above characteristics. New progress based on structural and MD simulation models, showing subtle differences from previous classic structures, has even allowed us to understand how the hERG channel functions and why it shows a higher affinity to some specific drugs.

Compared with drugs that affect the maturation of hERG channel proteins to decrease the I_Kr_ current, drugs that bind directly to the amino acid sites of the hERG channel to change the gating kinetics result in a more rapid channel failure. As mentioned above, these direct effects may sometimes produce extremely serious or even fatal cellular or clinical phenotypes (Zequn et al., 2021).

Currently, although novel structural views and some MD simulations with high numerical accuracy, they still cannot solve all the known and unknown factors that affect the actual hERG kinetics determined by the inherent complexity of biofilm channels (Li et al., 2021). These are limited by the availability of the structure and the limitations of MD simulation. Unlike the costly data obtained from experiments, computer simulations with lower cost will allow for quantitative hypothesis testing of how altered hERG functions. However, under different conditions of simulations, such as time, temperature, force field, and algorithm selection of MD, the opposite results may generate. Therefore, only by combining the high-resolution structure, more accurate MD, and experimental results, detailed studies of the hERG channel gating enable the generation of biophysically accurate models of the hERG gating kinetics. Incorporation of these molecular-level models into cells and ultimately whole hearts that will permit more informed predictions of how the hERG channel functions under physiological or pathological conditions (Perry et al., 2015). And it will bring us a molecular perspective of channel gating state changes, and by targeting these key molecules it can produce corrective effects on the hERG channel, such as activation (Garg et al., 2013; Zangerl-Plessl et al., 2020), enhancement (Wu et al., 2014; Donovan et al., 2018), and allosteric regulation (Sala et al., 2016; van Veldhoven et al., 2021).

## References

[B1] Adaixo R. Harley C. A. Castro-Rodrigues A. F. Morais-Cabral J. H. (2013). Structural Properties of PAS Domains from the KCNH Potassium Channels. PloS one 8, e59265. 10.1371/journal.pone.0059265 23555008 PMC3598652

[B4] Barros F. de la Peña P. Domínguez P. Sierra L. M. Pardo L. A. (2020). The EAG Voltage-dependent K^+^ Channel Subfamily: Similarities and Differences in Structural Organization and Gating. Front. Pharmacol. 11, 411. 10.3389/fphar.2020.00411 32351384 PMC7174612

[B2] Barros F. Domínguez P. de la Peña P. (2012). Cytoplasmic Domains and Voltage-dependent Potassium Channel Gating. Front. Pharmacol. 3, 49. 10.3389/fphar.2012.00049 22470342 PMC3311039

[B3] Barros F. Pardo L. Domínguez P. Sierra L. de la Peña P. (2019). New Structures and Gating of Voltage-dependent Potassium (Kv) Channels and Their Relatives: a Multi-Domain and Dynamic Question. Int. J. Mol. Sci. 20, 248. 10.3390/ijms20020248 PMC6359393 30634573

[B5] Ben-Bassat A. Giladi M. Haitin Y. (2020). Structure of KCNH2 Cyclic Nucleotide-Binding Homology Domain Reveals a Functionally Vital Salt-Bridge. J. Gen. Physiol. 152, e201912505. 10.1085/jgp.201912505 32191791 PMC7141593

[B6] Brewer K. R. Kuenze G. Vanoye C. G. George A. L. Jr. Meiler J. Sanders C. R. (2020). Structures Illuminate Cardiac Ion Channel Functions in Health and in Long QT Syndrome. Front. Pharmacol. 11, 550. 10.3389/fphar.2020.00550 32431610 PMC7212895

[B7] Butler A. Helliwell M. V. Zhang Y. Hancox J. C. Dempsey C. E. (2019). An Update on the Structure of hERG. Front. Pharmacol. 10, 1572. 10.3389/fphar.2019.01572 32038248 PMC6992539

[B9] Chen J. Seebohm G. Sanguinetti M. C. (2002). Position of Aromatic Residues in the S6 Domain, Not Inactivation, Dictates Cisapride Sensitivity of HERG and Eag Potassium Channels. Proc. Natl. Acad. Sci. 99, 12461–12466. 10.1073/pnas.192367299 12209010 PMC129467

[B10] Cheng W. W. L. McCoy J. G. Thompson A. N. Nichols C. G. Nimigean C. M. (2011). Mechanism for Selectivity-Inactivation Coupling in KcsA Potassium Channels. Proc. Natl. Acad. Sci. 108, 5272–5277. 10.1073/pnas.1014186108 21402935 PMC3069191

[B11] Codding S. J. Johnson A. A. Trudeau M. C. (2020). Gating and Regulation of KCNH (ERG, EAG, and ELK) Channels by Intracellular Domains. Channels 14, 294–309. 10.1080/19336950.2020.1816107 32924766 PMC7515569

[B12] Colenso C. K. Sessions R. B. Zhang Y. H. Hancox J. C. Dempsey C. E. (2013). Interactions between Voltage Sensor and Pore Domains in a hERG K+ Channel Model from Molecular Simulations and the Effects of a Voltage Sensor Mutation. J. Chem. Inf. Model. 53, 1358–1370. 10.1021/ci4000739 23672495

[B13] Corbin-Leftwich A. Mossadeq S. M. Ha J. Ruchala I. Le A. H. N. Villalba-Galea C. A. (2016). Retigabine Holds KV7 Channels Open and Stabilizes the Resting Potential. J. Gen. Physiol. 147, 229–241. 10.1085/jgp.201511517 26880756 PMC4772374

[B14] Cordero-Morales J. F. Cuello L. G. Zhao Y. Jogini V. Cortes D. M. Roux B. (2006). Molecular Determinants of Gating at the Potassium-Channel Selectivity Filter. Nat. Struct. Mol. Biol. 13, 311–318. 10.1038/nsmb1069 16532009

[B15] Cubeddu L. (2016). Drug-induced Inhibition and Trafficking Disruption of Ion Channels: Pathogenesis of QT Abnormalities and Drug-Induced Fatal Arrhythmias. Curr. Cardiol. Rev. 12, 141–154. 10.2174/1573403x12666160301120217 26926294 PMC4861943

[B16] Curran M. E. Splawski I. Timothy K. W. Vincen G. M. Green E. D. Keating M. T. (1995). A Molecular Basis for Cardiac Arrhythmia: HERG Mutations Cause Long QT Syndrome. Cell 80, 795–803. 10.1016/0092-8674(95)90358-5 7889573

[B17] de la Peña P. Domínguez P. Barros F. (2018). Gating Mechanism of Kv11.1 (hERG) K+ Channels without Covalent Connection between Voltage Sensor and Pore Domains. Pflugers Arch. - Eur. J. Physiol. 470, 517–536. 10.1007/s00424-017-2093-9 29270671 PMC5805800

[B18] Dehghani-Samani A. Madreseh-Ghahfarokhi S. Dehghani-Samani A. (2019). Mutations of Voltage-Gated Ionic Channels and Risk of Severe Cardiac Arrhythmias. Acta Cardiol. Sin 35, 99–110. 10.6515/ACS.201903_35(2).20181028A 30930557 PMC6434417

[B19] Delisle B. P. Anson B. D. Rajamani S. January C. T. (2004). Biology of Cardiac Arrhythmias. Circ. Res. 94, 1418–1428. 10.1161/01.res.0000128561.28701.ea 15192037

[B20] DeMarco K. R. Bekker S. Vorobyov I. (2019). Challenges and Advances in Atomistic Simulations of Potassium and Sodium Ion Channel Gating and Permeation. J. Physiol. 597, 679–698. 10.1113/jp277088 30471114 PMC6355641

[B21] Dickson C. J. Velez-Vega C. Duca J. S. (2020). Revealing Molecular Determinants of hERG Blocker and Activator Binding. J. Chem. Inf. Model. 60, 192–203. 10.1021/acs.jcim.9b00773 31880933

[B22] Dierich M. Evers S. Wilke B. U. Leitner M. G. (2018). Inverse Modulation of Neuronal K12.1 and K11.1 Channels by 4-Aminopyridine and NS1643. Front. Mol. Neurosci. 11, 11. 10.3389/fnmol.2018.00011 29440988 PMC5797642

[B23] Donovan B. T. Bandyopadhyay D. Duraiswami C. Nixon C. J. Townsend C. Y. Martens S. F. (2018). Discovery and Electrophysiological Characterization of SKF-32802: A Novel hERG Agonist Found through a Large-Scale Structural Similarity Search. Eur. J. Pharmacol. 818, 306–327. 10.1016/j.ejphar.2017.10.015 29050968

[B24] Egashira T. Yuasa S. Suzuki T. Aizawa Y. Yamakawa H. Matsuhashi T. (2012). Disease Characterization Using LQTS-specific Induced Pluripotent Stem Cells. Cardiovasc. Res. 95, 419–429. 10.1093/cvr/cvs206 22739119

[B25] Fernández-Mariño A. I. Harpole T. J. Oelstrom K. Delemotte L. Chanda B. (2018). Gating Interaction Maps Reveal a Noncanonical Electromechanical Coupling Mode in the Shaker K+ Channel. Nat. Struct. Mol. Biol. 25, 320–326. 10.1038/s41594-018-0047-3 29581567 PMC6170002

[B26] Flood E. Boiteux C. Lev B. Vorobyov I. Allen T. W. (2019). Atomistic Simulations of Membrane Ion Channel Conduction, Gating, and Modulation. Chem. Rev. 119, 7737–7832. 10.1021/acs.chemrev.8b00630 31246417

[B27] Forrest L. R. Tang C. L. Honig B. (2006). On the Accuracy of Homology Modeling and Sequence Alignment Methods Applied to Membrane Proteins. Biophysical J. 91, 508–517. 10.1529/biophysj.106.082313 PMC1483079 16648166

[B28] Garg V. Stary-Weinzinger A. Sanguinetti M. C. (2013). ICA-105574 Interacts with a Common Binding Site to Elicit Opposite Effects on Inactivation Gating of EAG and ERG Potassium Channels. Mol. Pharmacol. 83, 805–813. 10.1124/mol.112.084384 23319419 PMC3608434

[B29] Gianulis E. C. Liu Q. Trudeau M. C. (2013). Direct Interaction of Eag Domains and Cyclic Nucleotide-Binding Homology Domains Regulate Deactivation Gating in hERG Channels. J. Gen. Physiol. 142, 351–366. 10.1085/jgp.201310995 24043860 PMC3787778

[B30] Goodchild S. J. Macdonald L. C. Fedida D. (2015). Sequence of Gating Charge Movement and Pore Gating in HERG Activation and Deactivation Pathways. Biophysical J. 108, 1435–1447. 10.1016/j.bpj.2015.02.014 PMC4375626 25809256

[B31] Gustina A. S. Trudeau M. C. (2012). HERG Potassium Channel Regulation by the N-Terminal Eag Domain. Cell Signal. 24, 1592–1598. 10.1016/j.cellsig.2012.04.004 22522181 PMC4793660

[B32] Gustina A. S. Trudeau M. C. (2013). The Eag Domain Regulates hERG Channel Inactivation Gating via a Direct Interaction. J. Gen. Physiol. 141, 229–241. 10.1085/jgp.201210870 23319729 PMC3557309

[B33] He S. Moutaoufik M. T. Islam S. Persad A. Wu A. Aly K. A. (2020). HERG Channel and Cancer: A Mechanistic Review of Carcinogenic Processes and Therapeutic Potential. Biochim. Biophys. Acta Rev. Cancer 1873, 188355. 10.1016/j.bbcan.2020.188355 32135169

[B34] Heer F. T. Posson D. J. Wojtas-Niziurski W. Nimigean C. M. Bernèche S. (2017). Mechanism of Activation at the Selectivity Filter of the KcsA K Channel. Elife 6. e25844. 10.7554/elife.25844 28994652 PMC5669632

[B35] Helliwell M. V. Zhang Y. El Harchi A. Du C. Hancox J. C. Dempsey C. E. (2018). Structural Implications of hERG K+ Channel Block by a High-Affinity Minimally Structured Blocker. J. Biol. Chem. 293, 7040–7057. 10.1074/jbc.ra117.000363 29545312 PMC5936838

[B36] Herzik M. A. Jr. Fraser J. S. Lander G. C. (2019). A Multi-Model Approach to Assessing Local and Global Cryo-EM Map Quality. Structure 27, 344–358.e3. 10.1016/j.str.2018.10.003 30449687 PMC6365196

[B37] Hull C. M. Sokolov S. Van Slyke A. C. Claydon T. W. (2014). Regional Flexibility in the S4-S5 Linker Regulates hERG Channel Closed-State Stabilization. Pflugers Arch. - Eur. J. Physiol. 466, 1911–1919. 10.1007/s00424-013-1431-9 24407947

[B38] Jekhmane S. Medeiros-Silva J. Li J. Kümmerer F. Müller-Hermes C. Baldus M. (2019). Shifts in the Selectivity Filter Dynamics Cause Modal Gating in K(+) Channels. Nat. Commun. 10, 123. 10.1038/s41467-018-07973-6 30631074 PMC6328603

[B39] Jensen M. O. Jogini V. Borhani D. W. Leffler A. E. Dror R. O. Shaw D. E. (2012). Mechanism of Voltage Gating in Potassium Channels. Science 336, 229–233. 10.1126/science.1216533 22499946

[B40] Kalstrup T. Blunck R. (2018). S4-S5 Linker Movement during Activation and Inactivation in Voltage-Gated K+ Channels. Proc. Natl. Acad. Sci. USA 115, E6751–E6759. 10.1073/pnas.1719105115 29959207 PMC6055142

[B41] Ke Y. Hunter M. J. Ng C. A. Perry M. D. Vandenberg J. I. (2014). Role of the Cytoplasmic N-Terminal Cap and Per-Arnt-Sim (PAS) Domain in Trafficking and Stabilization of Kv11.1 Channels. J. Biol. Chem. 289, 13782–13791. 10.1074/jbc.m113.531277 24695734 PMC4022852

[B42] Kopec W. Rothberg B. S. de Groot B. L. (2019). Molecular Mechanism of a Potassium Channel Gating through Activation Gate-Selectivity Filter Coupling. Nat. Commun. 10, 5366. 10.1038/s41467-019-13227-w 31772184 PMC6879586

[B43] Kume S. Shimomura T. Tateyama M. Kubo Y. (2018). Two Mutations at Different Positions in the CNBH Domain of the hERG Channel Accelerate Deactivation and Impair the Interaction with the EAG Domain. J. Physiol. 596, 4629–4650. 10.1113/jp276208 30086184 PMC6166088

[B45] Labro A. J. Priest M. F. Lacroix J. J. Snyders D. J. Bezanilla F. (2015). Kv3.1 Uses a Timely Resurgent K(+) Current to Secure Action Potential Repolarization. Nat. Commun. 6, 10173. 10.1038/ncomms10173 26673941 PMC4703866

[B46] Labro A. J. Cortes D. M. Tilegenova C. Cuello L. G. (2018). Inverted Allosteric Coupling between Activation and Inactivation gates in K+ Channels. Proc. Natl. Acad. Sci. USA 115, 5426–5431. 10.1073/pnas.1800559115 29735651 PMC6003467

[B44] Labro A. J. Lacroix J. J. Villalba-Galea C. A. Snyders D. J. Bezanilla F. (2012). Molecular Mechanism for Depolarization-Induced Modulation of Kv Channel Closure. J. Gen. Physiol. 140, 481–493. 10.1085/jgp.201210817 23071266 PMC3483114

[B47] LeMasurier M. Heginbotham L. Miller C. (2001). Kcsa. J. Gen. Physiol. 118, 303–314. 10.1085/jgp.118.3.303 11524460 PMC2229506

[B49] Li J. Shen R. Reddy B. Perozo E. Roux B. (2021). Mechanism of C-type Inactivation in the hERG Potassium Channel. Sci. Adv. 7. eabd6203. 10.1126/sciadv.abd6203 33514547 PMC7846155

[B48] Li K. Jiang Q. Bai X. Yang Y.-F. Ruan M.-Y. Cai S.-Q. (2017). Tetrameric Assembly of K + Channels Requires ER-Located Chaperone Proteins. Mol. Cel. 65, 52–65. 10.1016/j.molcel.2016.10.027 27916661

[B50] Lörinczi É. Gómez-Posada J. C. de la Peña P. Tomczak A. P. Fernández-Trillo J. Leipscher U. (2015). Voltage-dependent Gating of KCNH Potassium Channels Lacking a Covalent Link between Voltage-Sensing and Pore Domains. Nat. Commun. 6, 6672. 10.1038/ncomms7672 25818916 PMC4389246

[B51] Malak O. A. Es-Salah-Lamoureux Z. Loussouarn G. (2017). hERG S4-S5 Linker Acts as a Voltage-dependent Ligand that Binds to the Activation Gate and Locks it in a Closed State. Scientific Rep. 7, 113. 10.1038/s41598-017-00155-2 PMC5427910 28273916

[B52] Malak O. A. Gluhov G. S. Grizel A. V. Kudryashova K. S. Sokolova O. S. Loussouarn G. (2019). Voltage-dependent Activation in EAG Channels Follows a Ligand-Receptor rather Than a Mechanical-Lever Mechanism. J. Biol. Chem. 294, 6506–6521. 10.1074/jbc.ra119.007626 30808709 PMC6484144

[B53] Männikkö R. Pandey S. Larsson H. P. Elinder F. (2005). Hysteresis in the Voltage Dependence of HCN Channels: Conversion between Two Modes Affects Pacemaker Properties. J. Gen. Physiol. 125, 305–326. 10.1085/jgp.200409130 15710913 PMC2234019

[B54] Mehta A. Ramachandra C. J. A. Singh P. Chitre A. Lua C. H. Mura M. (2018). Identification of a Targeted and Testable Antiarrhythmic Therapy for Long-QT Syndrome Type 2 Using a Patient-specific Cellular Model. Eur. Heart J. 39, 1446–1455. 10.1093/eurheartj/ehx394 29020304

[B55] Mitcheson J. S. Chen J. Lin M. Culberson C. Sanguinetti M. C. (2000). A Structural Basis for Drug-Induced Long QT Syndrome. Proc. Natl. Acad. Sci. 97, 12329–12333. 10.1073/pnas.210244497 11005845 PMC17341

[B56] Morais-Cabral J. H. Robertson G. A. (2015). The Enigmatic Cytoplasmic Regions of KCNH Channels. J. Mol. Biol. 427, 67–76. 10.1016/j.jmb.2014.08.008 25158096 PMC4277939

[B57] Muskett F. W. Thouta S. Thomson S. J. Bowen A. Stansfeld P. J. Mitcheson J. S. (2011). Mechanistic Insight into Human Ether-À-Go-Go-Related Gene (hERG) K+ Channel Deactivation Gating from the Solution Structure of the EAG Domain. J. Biol. Chem. 286, 6184–6191. 10.1074/jbc.m110.199364 21135103 PMC3057830

[B58] Ng C. A. Hunter M. J. Perry M. D. Mobli M. Ke Y. Kuchel P. W. (2011). The N-Terminal Tail of hERG Contains an Amphipathic α-helix that Regulates Channel Deactivation. PloS one 6, e16191. 10.1371/journal.pone.0016191 21249148 PMC3020963

[B59] Olcese R. Latorre R. Toro L. Bezanilla F. Stefani E. (1997). Correlation between Charge Movement and Ionic Current during Slow Inactivation in Shaker K+ Channels. J. Gen. Physiol. 110, 579–589. 10.1085/jgp.110.5.579 9348329 PMC2229383

[B60] Ono M. Burgess D. E. Schroder E. A. Elayi C. S. Anderson C. L. January C. T. (2020). Long QT Syndrome Type 2: Emerging Strategies for Correcting Class 2 KCNH2 (hERG) Mutations and Identifying New Patients. Biomolecules 10, 1144. 10.3390/biom10081144 PMC7464307 32759882

[B61] Pan A. C. Cuello L. G. Perozo E. Roux B. (2011). Thermodynamic Coupling between Activation and Inactivation Gating in Potassium Channels Revealed by Free Energy Molecular Dynamics Simulations. J. Gen. Physiol. 138, 571–580. 10.1085/jgp.201110670 22124115 PMC3226968

[B62] Perry M. de Groot M. J. Helliwell R. Leishman D. Tristani-Firouzi M. Sanguinetti M. C. (2004). Structural Determinants of HERG Channel Block by Clofilium and Ibutilide. Mol. Pharmacol. 66, 240–249. 10.1124/mol.104.000117 15266014

[B64] Perry M. D. Ng C.-A. Mangala M. M. Ng T. Y. M. Hines A. D. Liang W. (2020). Pharmacological Activation of IKr in Models of Long QT Type 2 Risks Overcorrection of Repolarization. Cardiovasc. Res. 116, 1434–1445. 10.1093/cvr/cvz247 31628797

[B63] Perry M. D. Ng C.-A. Mann S. A. Sadrieh A. Imtiaz M. Hill A. P. (2015). Getting to the Heart of hERG K+channel Gating. J. Physiol. 593, 2575–2585. 10.1113/jp270095 25820318 PMC4500344

[B65] Priest M. F. Lacroix J. J. Villalba-Galea C. A. Bezanilla F. (2013). S3-S4 Linker Length Modulates the Relaxed State of a Voltage-Gated Potassium Channel. Biophysical J. 105, 2312–2322. 10.1016/j.bpj.2013.09.053 PMC3838747 24268143

[B66] Roden D. M. Yang T. (2005). Protecting the Heart against Arrhythmias: Potassium Current Physiology and Repolarization reserve. Circulation 112, 1376–1378. 10.1161/circulationaha.105.562777 16145010

[B67] Sala L. Yu Z. Ward‐van Oostwaard D. Veldhoven J. P. Moretti A. Laugwitz K. L. (2016). A New hERG Allosteric Modulator Rescues Genetic and Drug‐induced Long‐ QT Syndrome Phenotypes in Cardiomyocytes from Isogenic Pairs of Patient Induced Pluripotent Stem Cells. EMBO Mol. Med. 8, 1065–1081. 10.15252/emmm.201606260 27470144 PMC5009811

[B68] Schewe M. Sun H. Mert Ü. Mackenzie A. Pike A. C. W. Schulz F. (2019). A Pharmacological Master Key Mechanism that Unlocks the Selectivity Filter Gate in K+channels. Science 363, 875–880. 10.1126/science.aav0569 30792303 PMC6982535

[B69] Schmitt N. Grunnet M. Olesen S.-P. (2014). Cardiac Potassium Channel Subtypes: New Roles in Repolarization and Arrhythmia. Physiol. Rev. 94, 609–653. 10.1152/physrev.00022.2013 24692356

[B70] Schönherr R. Heinemann S. H. (1996). Molecular Determinants for Activation and Inactivation of HERG, a Human Inward Rectifier Potassium Channel. J. Physiol. 493 (Pt 3), 635–642. 10.1113/jphysiol.1996.sp021410 8799887 PMC1159013

[B72] Schwartz P. J. Ackerman M. J. (2013). The Long QT Syndrome: a Transatlantic Clinical Approach to Diagnosis and Therapy. Eur. Heart J. 34, 3109–3116. 10.1093/eurheartj/eht089 23509228

[B74] Schwartz P. J. Gnecchi M. Dagradi F. Castelletti S. Parati G. Spazzolini C. (2019). From Patient-specific Induced Pluripotent Stem Cells to Clinical Translation in Long QT Syndrome Type 2. Eur. Heart J. 40, 1832–1836. 10.1093/eurheartj/ehz023 30753398

[B73] Schwartz P. J. Woosley R. L. (2016). Predicting the Unpredictable. J. Am. Coll. Cardiol. 67, 1639–1650. 10.1016/j.jacc.2015.12.063 27150690

[B76] Shi Y. P. Thouta S. Claydon T. W. (2020). Modulation of hERG K^+^ Channel Deactivation by Voltage Sensor Relaxation. Front. Pharmacol. 11, 139. 10.3389/fphar.2020.00139 32184724 PMC7059196

[B75] Shi Y. P. Thouta S. Cheng Y. M. Claydon T. W. (2019). Extracellular Protons Accelerate hERG Channel Deactivation by Destabilizing Voltage Sensor Relaxation. J. Gen. Physiol. 151, 231–246. 10.1085/jgp.201812137 30530765 PMC6363419

[B77] Skinner J. R. Winbo A. Abrams D. Vohra J. Wilde A. A. (2019). Channelopathies that Lead to Sudden Cardiac Death: Clinical and Genetic Aspects. Heart Lung Circ. 28, 22–30. 10.1016/j.hlc.2018.09.007 30389366

[B79] Smith J. L. Anderson C. L. Burgess D. E. Elayi C. S. January C. T. Delisle B. P. (2016). Molecular Pathogenesis of Long QT Syndrome Type 2. J. arrhythmia 32, 373–380. 10.1016/j.joa.2015.11.009 PMC5063260 27761161

[B78] Smith P. L. Baukrowitz T. Yellen G. (1996). The Inward Rectification Mechanism of the HERG Cardiac Potassium Channel. Nature 379, 833–836. 10.1038/379833a0 8587608

[B80] Spector P. S. Curran M. E. Zou A. Keating M. T. Sanguinetti M. C. (1996). Fast Inactivation Causes Rectification of the IKr Channel. J. Gen. Physiol. 107, 611–619. 10.1085/jgp.107.5.611 8740374 PMC2217012

[B81] Stansfeld P. J. Grottesi A. Sands Z. A. Sansom M. S. P. Gedeck P. Gosling M. (2008). Insight into the Mechanism of Inactivation and pH Sensitivity in Potassium Channels from Molecular Dynamics Simulations. Biochemistry 47, 7414–7422. 10.1021/bi800475j 18558719

[B82] Thouta S. Hull C. M. Shi Y. P. Sergeev V. Young J. Cheng Y. M. (2017). Stabilization of the Activated hERG Channel Voltage Sensor by Depolarization Involves the S4-S5 Linker. Biophysical J. 112, 300–312. 10.1016/j.bpj.2016.12.021 PMC5266146 28122216

[B83] Tilegenova C. Cortes D. M. Cuello L. G. (2017). Hysteresis of KcsA Potassium Channel's Activation- Deactivation Gating Is Caused by Structural Changes at the Channel's Selectivity Filter. Proc. Natl. Acad. Sci. USA 114, 3234–3239. 10.1073/pnas.1618101114 28265056 PMC5373385

[B84] Tristani-Firouzi M. Chen J. Sanguinetti M. C. (2002). Interactions between S4-S5 Linker and S6 Transmembrane Domain Modulate Gating of HERG K+ Channels. J. Biol. Chem. 277, 18994–19000. 10.1074/jbc.m200410200 11864984

[B85] van Noord C. Eijgelsheim M. Stricker B. H. C. (2010). Drug- and Non-drug-associated QT Interval Prolongation. Br. J. Clin. Pharmacol. 70, 16–23. 10.1111/j.1365-2125.2010.03660.x 20642543 PMC2909803

[B86] van Veldhoven J. P. D. Campostrini G. van Gessel C. J. E. Ward-van Oostwaard D. Liu R. Mummery C. L. (2021). Targeting the Kv11.1 (hERG) Channel with Allosteric Modulators. Synthesis and Biological Evaluation of Three Novel Series of LUF7346 Derivatives. Eur. J. Med. Chem. 212, 113033. 10.1016/j.ejmech.2020.113033 33261899

[B88] Vandenberg J. I. Perozo E. Allen T. W. (2017). Towards a Structural View of Drug Binding to hERG K + Channels. Trends Pharmacological Sciences 38, 899–907. 10.1016/j.tips.2017.06.004 PMC6658208 28711156

[B87] Vandenberg J. I. Perry M. D. Perrin M. J. Mann S. A. Ke Y. Hill A. P. (2012). hERG K+ Channels: Structure, Function, and Clinical Significance. Physiol. Rev. 92, 1393–1478. 10.1152/physrev.00036.2011 22988594

[B89] Villalba-Galea C. A. (2017). Hysteresis in Voltage-Gated Channels. Channels 11, 140–155. 10.1080/19336950.2016.1243190 27689426 PMC5398603

[B91] Wang D. T. Hill A. P. Mann S. A. Tan P. S. Vandenberg J. I. (2011). Mapping the Sequence of Conformational Changes Underlying Selectivity Filter Gating in the Kv11.1 Potassium Channel. Nat. Struct. Mol. Biol. 18, 35–41. 10.1038/nsmb.1966 21170050

[B90] Wang W. MacKinnon R. (2017). Cryo-EM Structure of the Open Human Ether-À-Go-Go -Related K + Channel hERG. Cell 169, 422–430.e10. 10.1016/j.cell.2017.03.048 28431243 PMC5484391

[B92] Whicher J. R. MacKinnon R. (2016). Structure of the Voltage-Gated K+ Channel Eag1 Reveals an Alternative Voltage Sensing Mechanism. Science 353, 664–669. 10.1126/science.aaf8070 27516594 PMC5477842

[B93] Wilson S. L. Dempsey C. E. Hancox J. C. Marrion N. V. (2019). Identification of a Proton Sensor that Regulates Conductance and Open Time of Single hERG Channels. Scientific Rep. 9, 19825. 10.1038/s41598-019-56081-y PMC6934679 31882846

[B94] Witchel H. J. Hancox J. C. J. C. Pharmacology E. (2001). Physiology. Familial and Acquired Long QT Syndrome and the Cardiac Rapid Delayed. Rectifier Potassium Curr. 27, 753–766. 10.1046/j.1440-1681.2000.03337.x 11022966

[B95] Wu W. Sachse F. B. Gardner A. Sanguinetti M. C. (2014). Stoichiometry of Altered hERG1 Channel Gating by Small Molecule Activators. J. Gen. Physiol. 143, 499–512. 10.1085/jgp.201311038 24638994 PMC3971662

[B96] Xu Y. McDermott A. E. (2019). Inactivation in the Potassium Channel KcsA. J. Struct. Biol. X 3, 100009. 10.1016/j.yjsbx.2019.100009 32647814 PMC7337057

[B97] Yan M. Fan P. Shi Y. Feng L. Wang J. Zhan G. (2016). Stereoselective Blockage of Quinidine and Quinine in the hERG Channel and the Effect of Their Rescue Potency on Drug-Induced hERG Trafficking Defect. Int. J. Mol. Sci. 17, 1648. 10.3390/ijms17101648 PMC5085681 27690007

[B98] Zangerl-Plessl E.-M. Berger M. Drescher M. Chen Y. Wu W. Maulide N. (2020). Toward a Structural View of hERG Activation by the Small-Molecule Activator ICA-105574. J. Chem. Inf. Model. 60, 360–371. 10.1021/acs.jcim.9b00737 31877041

[B99] Zequn Z. Yujia W. Dingding Q. Jiangfang L. (2021). Off-label Use of Chloroquine, Hydroxychloroquine, Azithromycin and Lopinavir/ritonavir in COVID-19 Risks Prolonging the QT Interval by Targeting the hERG Channel. Eur. J. Pharmacol. 893, 173813. 10.1016/j.ejphar.2020.173813 33345848 PMC7746509

[B100] Zhang K.-p. Yang B.-f. Li B.-x. (2014). Translational Toxicology and rescue Strategies of the hERG Channel Dysfunction: Biochemical and Molecular Mechanistic Aspects. Acta Pharmacol. Sin 35, 1473–1484. 10.1038/aps.2014.101 25418379 PMC4261120

[B101] Zhang X. C. Yang H. Liu Z. Sun F. (2018). Thermodynamics of Voltage-Gated Ion Channels. Biophys. Rep. 4, 300–319. 10.1007/s41048-018-0074-y 30596139 PMC6276078

[B102] Zhang Y. Dempsey C. E. Hancox J. C. (2020). Electrophysiological Characterization of the Modified hERG Potassium Channel Used to Obtain the First Cryo-EM hERG Structure. Physiol. Rep. 8, e14568. 10.14814/phy2.14568 33091232 PMC7580876

